# Why Do We Give Alcoholic Stimulants in Shock?

**Published:** 1867-12

**Authors:** D. W. Young

**Affiliations:** Aurora, Ill.


CHICAGO MEDICAL EXAMINER.

N. S. DAVIS, M.D., Editor.

VOL. VIII. DECEMBER, 1867. NO. 12.

### Original Contributions.

ARTICLE XLIX.

### WHY DO WE GIVE ALCOHOLIC STIMULANTS IN SHOCK?

By D. W. YOUNG, M.D., Aurora, Ill.

Read before the Fox River Valley Medical Association, October 7th, 1867.

Mr. President and Gentlemen:

The above is an important question, both to the profession and the public; is important from its moral as well as its physical relations to society. However, I do not propose to constitute myself a reformer and treat of its immoral bearings upon society, I will leave that to those who make more pretensions in that direction; still, in my judgment, it behooves us all, as a profession, who should be conservators of public morals as well as public health, to study well the reasons that induce us, as a profession, to insist and persist in the sanction and recommendation of an article which is constantly producing such terribly degrading influences upon human society. I cannot help thinking that unless upon a thorough examination *we find it imperative*, we are not justifiable in lending our influence and sanction to its so general use as a medicine. The opinion that alcoholic liquors afford to the human system a stimulus, which, if not absolutely necessary and essential to its well-being, is, nevertheless, beneficial, by promoting in the several organs a vigorous and healthful exercise of their respective functions, and by enabling them, thus, to resist more effectually the various disturbing agencies to which they are daily subjected, is one that has been long entertained, and of the correctness of which a large portion of the public still entertains a firm conviction. Most undoubtedly, to the influence of this opinion may *and must* be ascribed much of the intemperance which has prevailed in the world; and it, even now, presents a formidable barrier to the success of every effort at reform, in respect to the use of intoxicating liquors as a beverage.

We all know that the practice of giving alcoholic stimulants in all cases of shock, whether physical or mental, has become so common, that not only all physicians, but all the public, fly to the whiskey and brandy bottles as the elixir of life—the great restorers of health and animation. So long as this opinion prevails, every effort to stay the progress of intemperance, with its attendant evils, disease, poverty, insanity, and crime, will of necessity be unavailing.

Is this practice in conformity with the best known medical facts—facts of undoubted observation and experience? Is it based upon scientific medical investigation; or is it simply custom, handed down to us from the dark ages of the profession? To try and answer these questions is the object of this paper. To investigate this subject understandingly, it will be necessary to inquire what shock is; what the physical condition that produces it; what the lesion to be remedied; also, what the known or supposed physiological action or effects of alcoholic stimulants are upon the human system.

Prof. Dunglison says:—Shock is a "sudden or instantaneous depression of organic, nervous, or vital power, often with more or less perturbation of body and mind, passing either into reaction or into fatal sinking, occasioned by the nature, severity, or extent of injury, or by an overwhelming moral calamity."

Prof. Gross, in his system of surgery, says:—"Shock may be defined to be a depression of the vital powers, induced suddenly by external injury, and essentially dependent upon a loss of innervation. It bears, in effect, the same relation to the nervous system, that syncope does to the vascular; in the one case, the result is caused by a diminution of blood; in the other, by a diminution of the nervous fluid; in both, the consequence is, more or less prostration, with perturbation of body and mind, extremely variable both as to intensity and duration. When nervous shock is severe, it may instantaneously terminate in death, as so often happens in falls and blows upon the head; more generally, however, after having continued an indefinite period, it passes into reaction, the powers of life gradually coming up as the different organs and the general system regain their nervous fluid. The most severe and fatal cases of shock are, generally, those that supervene upon direct injury to the great nervous-centres, as the brain and spinal cord. No less disastrous effects occasionally succeed blows upon the epigastric region, in consequence of the violence thus inflicted upon the solar plexus of nerves. The blood has long been known by physiologists, as the vital fluid, so necessary has its integrity always been regarded to the well-being of the system and maintainance of healthy action. But, certainly, it is not the only fluid entitled to this distinction; the nervous fluid is both more subtle and more important as a life preserver. When the blood flows away in a mighty and overwhelming torrent, the person dies, and life is then said to be destroyed, as it certainly is, by the excessive sanguineous drainage. But in shock, the same thing may happen, and yet the body be literally surcharged with blood, not a single drop, perhaps, having been spilled in the accident causing the fatal result. Thus, of the two fluids, the nervous is in every respect the more important, because the more essential to life; and its disturbance is, therefore, a more frequent cause of death.

"There is no sensible practitioner who has not occasion daily to lament, in the exercise of his profession, his want of knowledge of the functions of the nervous system, and I feel sure that cases of disease and accidents are constantly permitted to slip through our hands, simply because of our forgetfulness that there is such a thing as nervous fluid. No one is unmindful that a patient has blood, that a certain quantity of this fluid is necessary to the maintainance of health and life, and that, like the solids, it is subject to a thousand diseases, often of themselves sufficient to cause death. Unfortunately, we can never acquire any intimate knowledge of an agent so subtle as the nervous fluid; like the electric or galvanic, which it is supposed to resemble, we can know it only by its effects."

Copeland defines shock to be a sudden sinking of vitality; vital depression; nervous shock; nervous depression; fatal sinking. He makes five divisions or classes:

*First.* Where the shock may be altogether and simply a vital one, as where it is produced by a violent blow on the epigastrium, occasioning concussion of the solar ganglion.

*Second.* Where it may be associated with various nervous phenomena, as when a large nerve, or joint, or limb, is lacerated or severely injured, and the patient greatly alarmed.

*Third.* Where it may be complicated with, or rather characterized by, comatose sinking, as when the contusion, concussion, or blow affects the intimate organization and circulation of the brain.

*Fourth.* Where it may be so associated with the sinking, consequent upon losses of blood, as not to be distinguished from this cause, especially when the injury is such as occasions both shock and hemorrhage.

*Fifth.* Where the alarm or shock may be entirely a mental one, or that consisting entirely of the sudden effects of extremely depressing emotions on the action of the heart, or of the sudden and unexpected intelligence of distressing losses or events, whereby the nervous system is more or less shocked, the mental manifestations disturbed, and the functions of the heart and vital organs depressed and otherwise disordered.

He adds:—"It will thus be perceived that the injuries or *causes* occasioning shock may be divided into *five classes*, and that the effects they produce may present *five modified forms;* but although either of these may result from either class of causes, and although it is necessary to connect our observation of the phenomena, and our treatment of shock, with the particular cause of it, it is still more important, especially as regards the treatment, to mark the particular form and modification requiring our aid."

ERICHSEN says:—"The constitutional effects of shock consist in a disturbance of the functions of the circulatory, respiratory, and nervous systems, the harmony of the great organs of the body becoming disarranged. On the receipt of a severe injury, the sufferer becomes cold, faint, and trembling; the pulse small and fluttering; there is great mental depression and disquietude; the disturbed state of mind revealing itself in the countenance, and in incoherence of speech and thought; the surface becomes covered by a cold sweat; there is nausea, perhaps vomiting, and relaxation of the sphincters. This condition lasts for a variable period, its duration depending on the severity of the injury, and on the nervous susceptibility of the patient. In extreme cases, the depression of power characterizing shock may be so great as to terminate in death. In the great majority of instances, however, reaction comes on, and the disturbed balance in the system is gradually restored. Is this state the result of fear and nervous susceptibility, or is it occasioned directly by the physical lesion? In many cases, doubtless, the effect is entirely mental. Thus, persons have been frightened to death without any local injury or mischief. That there is a great difference in the mental fortitude of individuals, is notorious; some suffering excessive shock from the mere apprehension of injury, whilst others may be the subjects of the severest injury, and show but slight signs of suffering. If the injury be sudden and unexpected, the shock is usually greater. Where the feelings are roused, as in the heat of action, injuries often pass unnoticed by those who receive them. Hence, it is positively evident, that the state of mind at the time of the receipt of the injury materially influences its immediate effect on the constitution. There can be little doubt, however, that different individuals manifest very different degrees of susceptibility to pain, some having more acute sensation than others."

Thus, it will be seen that all these eminent surgeons and authors agree, and define shock to be a "*sudden or instantaneous diminution or depression of organic, nervous, or vital power.*"

All agree that it varies in different cases and persons, according to the severity and extent of the cause or the injury, and the particular temperament, physical and mental condition of the patient at the time of the receipt of the cause or the injury. All these various conditions and circumstances are admitted to have a modifying or controlling influence upon the malady. Why this is so, we are unable to explain; because we do not know what life is—our present limited knowledge of the science of life does not enable us to give an exhaustive definition. Beale, Hunter, Harvey, Aristotle, Abernethy, Muller, Prout, Carpenter, Hammer, and Virchow have all advanced extensive theories, have all written learnedly and guessed extensively, but the question still is, What is life?

The symptoms of shock are very similar in all persons, and from all causes. One receives a blow upon the head; another upon the epigastrium; another, upon the back; another, falls a distance; another, receives sad news, such as the death of friends, or the loss of property; still another, simply witnesses a frightful accident. All reveal the same or similar symptoms. All fall to the ground; become suddenly cold; are bathed in a cold sweat; with deathly paleness; a sunken or collaped countenance; irregular gasping and difficult breathing; are unable to move, speak, or think; frequently become convulsed; vomit; and, after a longer or shorter period, reaction or fatal sinking supervene, life either ceases, or healthy action is restored and life continues. This depressed condition of the organic, nervous, or vital powers we denominate shock, and the question now is, Are alcoholic stimulants the *sine qua non* to remedy this condition? Are we compelled to resort to this health, wealth, happiness, intellect, and life destroying agent to remedy the evil, to remove the shock?

Let us now carefully and candidly enquire what our own experience and the teachings and writings of our ablest and best physiologists and practitioners teach us upon this point. Learn from all this evidence, how well alcoholic stimulants are entitled to their present exalted position in the cure of this prostration. See if we *must* continue to sanction and recommend their use.

Griffith says:—"In small quantities, there is mere excitement; in large doses, much excitement, with delirium, confusion of intellect, followed by somnolency, nausea, and vomiting, and even coma and apoplexy."

Stille, in his Materia Medica, says, when speaking of its effects on animals:—"When the crural nerve of a frog is moistened with this liquid, the limb loses its power of motion; the same results, with *depressed* action of the heart, ensues when the whole limb is wet with alcohol. The pulsations of the heart soon cease under its application. Fish immediately lose their activity, in water containing but a small proportion of this liquid; and birds, according to Flourens, are deprived of sensation and voluntary motion, by a few drops of brandy. Injected into the veins, alcohol always produces symptoms of prostration, proportioned to the quantity used and the purity of the article.

"As early as 1679, Courten, Lanzani, and Baglivi, all showed that highly rectified spirits might prove instantly fatal, when employed in this manner, and that after death the blood *was always found coagulated in the heart and lungs.*"

Segalas "found that half an ounce, with four or five times its weight of water, injected into the crural or jugular vein of a dog, produced loss of motion, insensibility, abdominal respiration, and a *scarcely perceptible pulse.*"

Orfila "found that eight or ten drachms, injected into the cellular tissue of a dog, produced, first, vomiting of bilious matters, and death in three hours. On dissection, the only lesion discoverable, was coagulation of the blood in the limb operated upon, *and in the heart.*"

Dunglison says:—"It need scarcely be said, that the case must be bad indeed, in which the hopes of the practitioner are placed on the excitement which alcohol is capable of inducing. It may be a *question, indeed,* whether it be not calculated to to exhaust the slight amount of excitability still existing in the system."

Wood and Bache, in the United States Dispensatory, say: "As an article of daily use, alcoholic liquors produce the most deplorable consequences. Besides the moral degradation which they cause, their habitual use gives rise to dyspepsia, hypochondriasis, visceral obstructions, dropsy, paralysis, *and, not unfrequently, mania.*"

Copeland says:—"It should be recollected that the effects of spirits or other intoxicating liquors on the frame, will vary very much with the habits of the individual; with his state of body, especially as respects vascular plethora; and the existing condition of the stomach, chiefly as respects the presence of alimentary matters. In the larger proportion of cases, however, after a longer or shorter period of unusual mental vigor, nervous excitement, and increased action, varying according to the surrounding temperature, the brain becomes oppressed; the powers of voluntary motion, which are early impaired, fail entirely; the mental manifestations are suspended; and in the more severe cases, sensation is lost completely. In most cases and instances, this stage supervenes gradually; but any sudden exposure to cold will often induce it rapidly. The person feels drowsy, and appears to fall into a sound sleep; but it is discovered, when the attempt is made, that he cannot be aroused to consciousness by any effort, or, if it partially succeed, he is hardly sensible of surrounding objects, and immediately lapses into his former state, the limbs remain in whatever position they may be placed. The temperature of the head is generally above natural; but that of the extremities, and often of the surface generally, is *considerably lowered*, or but little altered or affected in the milder cases. The pulse, which was at first quick and excited, becomes feeble, small, and ultimately slow, and entirely wanting at the wrist. The respiration is usually infrequent, the separate acts of inspiration and expiration, particularly the former, occupying a very short time, and is wholly or chiefly abdominal. The breathing is often laborious in the most advanced states, and in those, the respirations are convulsive, the chest expanding by the rapid contractions of the associated muscles of respiration."

Williams, in his Principles of Medicine, says:—"Nor can we wonder at the pernicious effects, when we consider the weakened state of the function and structure which stimulating drinks induce, especially in the organs which they most directly affect, the stomach, the liver, the kidneys, the blood, the heart, and the brain."

All these eminent authors and teachers ascribe to alcohol, either as a primary or secondary effect, an enfeebled action of the heart and circulation. Besides these, we have the very valuable and interesting experiments and concise report of Prof. N. S. DAVIS, of Chicago, who called both the sphygmograph and thermometer to his aid. As his experiments are both very interesting and very instructive, I shall copy them entire:

"On the 6th day of April, 1867, four hours after dinner, when the functions were supposed to be undisturbed by digestion, and the man in good health, the temperature of his body was carefully noted by a delicately graduated thermometer, inserted under the tongue, with the lips closed around it; the rate of the pulse and its qualities, as indicated by the sphygmograph, were recorded at the same time. Four ounces of bourbon whiskey was then administered, diluted with sweetened water. The same observations, in regard to temperature and condition of pulse, were made and recorded every half hour, until two full hours had passed. Another series of observations, in all respects similar, were made on the 11th of April, except the whiskey, for which four ounces of sherry wine was substituted.

"At the commencement of the experiment, at 10.20 o'clock P.M., the temperature of the mouth was $98\frac{1}{4}$°, pulse 83 per minute. Then he took the four ounces of whiskey. At 11 o'clock P.M., half an hour after he had taken the whiskey, the temperature of the mouth was $97\frac{3}{4}$°, pulse 85 per minute. At 11.30 o'clock P.M., one hour after he had taken the whiskey, the temperature of his mouth was $97\frac{1}{2}$, pulse 89 per minute. At 12 o'clock P.M., one hour and a-half after the whiskey was taken, the temperature of his mouth was $97\frac{1}{2}$°, pulse 89 per minute. At 12.30 A.M., two hours after the whiskey had been administered, the temperature of his mouth was $97\frac{1}{2}$°, pulse 85 perminute."

Thus, it will be seen that in this experiment, when four ounces of bourbon whiskey were administered, the pulse increased from 83 to 89 beats per minute during the first hour, but decreased in number of beats per minute, from 89 to 85, during the second hour. The sphygmograph shows, most conclusively, that, notwithstanding, while the number of pulsations were increased from 83 to 89 per minute during the first hour, *the force of the heart and pulsations were weakened.* The heart evidently had less power to propel the blood through the arteries, *and a congestion of the venous radicles must ensue.* The same thing often takes place during venesection, the number of pulsations are increased per minute, but the power of the heart is reduced.

The second experiment made by Prof. DAVIS, differs but little from the first. It was instituted on the 11th day of April, 1867, and is as follows:—"At 10.15 o'clock P.M., three and a-half hours after dinner, the temperature of the mouth was tested with the thermometer, and found to be 97°, pulse 78 beats per minute. At 10.30 o'clock P.M., four ounces of pure, clear sherry wine were administered. At 11 o'clock P.M., half an hour after the wine was swallowed, the temperature of the mouth was $96\frac{3}{4}$°, pulse 75 per minute. At 11.30 o'clock P.M., one hour after the wine was taken, the temperature of the mouth was $96\frac{1}{2}$°, pulse 71 per minute. At 12.30, o'clock P.M., two hours after the wine had been drank, the temperature of the mouth was $96\frac{1}{2}$°, pulse 72 per minute." Thus, it will be seen that under the influence of four ounces of wine, the pulsations were reduced in number per minute, from 78 to 72—lost six beats in two hours. The sphygmograph again shows distinctly that the quality of the pulse was the same in kind in both experiments. *The force of the heart's action was reduced under the influence of both the wine and the whiskey.*

Dr. DAVIS, in commenting upon the results of his experiments, says—"It will be seen that each pulse expands the artery to a greater extent and more suddenly than before the alcoholic liquid was taken, and that the commencement of the contraction is equally more sudden, while the whole line becomes more wavy or irregular; thereby much resembling the *pulse lines* (as shown by the sphygmograph) when the arterial coats are weakened by fatty degeneration, or in such diseases as are accompanied by enfeebled capillary circulatlon, like typhus and typhoid fevers."

Here, I desire to add that this fact was very forcibly and strikingly illustrated by comparing them with some fifteen or twenty plates presented to the Illinois State Medical Society, at Springfield, by Prof. H. A. JOHNSON, of Chicago, who also presented the results of his experiments with the sphygmograph, in a large number of cases and different diseases. Prof. JOHNSON'S plates showing the *pulse lines*, as recorded by the sphygmograph, in a number of cases of typhoid fever and chronic diarrhœa, were apparently identical with those presented by Prof. DAVIS, as recorded by the sphygmograph in his experiments with alcoholic stimulants. This fact was very apparent, and was noticed and commented upon by numerous and various members of the association. Both sets of plates showed, most *unmistakably, enfeebled action of the heart*, notwithstanding the one was recorded while the patient was laboring under the influence of four ounces of bourbon whiskey, and the other after the patient had suffered several weeks under the depressing influence of typhoid fever and chronic diarrhœa.

Dr. LIONEL BEALE, Physician to King's College Hospital, in an article in *Braithwaite*, on Stimulation in Serious Cases of Acute Disease, says:—"I shall not discuss *how* stimulants, in very large quantity, influence disease, but shall, in conclusion, beg permission to direct attention to certain clinical facts which have been observed in many cases placed under the influence of large quantities of alcoholic stimulants (*eig teen ounces of brandy and upwards in twenty four hours*) the pulse was not increased, but diminished in frequency."

Let us next inquire how alcoholic stimulants affect the temperature of the body. So far as I can learn, but few really systematic scientific investigations have been made in this direction, and the proofs relied upon as proving the heat-producing power of alcohol, rest chiefly upon the sensation arising from its ingestion, and on its chemical constitution, which latter is considered, by those who support LIEBIG'S theory of animal heat, to furnish evidence of the effectiveness of alcoholic compounds as sources of heat, when introduced into the living organism.

It has been, somewhat loosely and gratuitously, assumed that the agent in question exercises a powerful influence on the production of animal heat, this assumption being based principally on the sensation of warmth experienced in our own persons, after taking alcoholic liquors. We know, however, that our sensations are extremely fallacious guides in all matters pertaining to temperature, and that, therefore, in this case as in others, but little reliance can be placed on their indications. I am fully aware that the great majority of authorities are in favor of the opinion that alcohol is a powerful promoter of animal heat. Many patient, honest, intelligent, and competent experimenters, however, deny this, and, in my judgment, give reasons and results which are at least entitled to our candid consideration. As one means of arriving at the truth on this point, we must examine more particularly into the influence of the introduction of alcohol into the blood, upon the respiratory process. For our knowledge upon this point, we are largely indebted to the experiments of Dr. PROUT and Dr. VIERORDT. The former, states that alcohol and all liquors containing it, which he had tried, have the remarkable power of diminishing the quantity of carbonic acid gas in the expired air, much more than anything else which he had made the subject of experiments; this effect being most decided when the liquor was taken upon an empty stomach. Dr. VIERORDT fully confirms Dr. PROUT'S observations; having found that in four experiments, the percentage of carbonic acid fell, after from a half to a whole bottle of wine had been taken, from 4.54 to 4.01; and that this effect lasted between one and two hours. He further found, that when he drank wine with his dinner, the usual increase in the percentage of carbonic acid expired after a full meal did not take place. These facts are of great importance.

Thus, then, there are clear indications that, when thus present in the blood with other materials which ought to be excreted, alcohol exerts an injurious influence, by retarding their combustion. This it will do in two ways:—first, by taking their place as the more readily combustible material; and, secondly, in virtue of the antiseptic influence which it exerts upon other substances, preventing or retarding chemical changes in them. That such is the case, we have at least strong proof, as appears from the experiments of BOUCHARDAT, who found that, when alcohol is introduced into the system in excess, the blood in the arteries presents the aspect of venous blood, clearly showing that it has been prevented from undergoing the proper oxygenating process.

The experiments of Dr. PROUT afford strong additional support to this conclusion; for he observed that no sooner had the effects of the alcohol passed off, than the amount of carbonic acid exhaled *rises much above* the natural standard, thus giving, it would seem, unequivocal evidence of the previous abnormal retention of carbonaceous matter in the system.

From the foregoing considerations, then, we may conclude that the effects of alcohol as a heat-producing material, at best, could only be advantageously experienced when the blood does not contain a supply of other matters waiting for removal by the respiratory process. Is it not the case that in all cases of shock, the respiratory process is but imperfectly performed, and that, as a necessary consequence, there must be an excess of carbonic acid retained in the blood? If such is the fact, can it be a logical procedure to administer such articles as will still further increase the already sedative influence of an excess of venous blood?

Dr. JOHN DAVY found that wine, so from increasing the temperature of his body, caused, on the contrary, a very visible diminution of its heat; and, moreover, that the diminution was proportioned to the quantity of wine taken—the greater the quantity, the more marked being the decrease of temperature.

Dr. J. D. HOOKER, who accompanied Sir JAMES ROSS in his Antarctic expedition, also denies the heat-producing properties of alcohol, and says, "that, even if it is capable of warming the central portions of the body, it is incapable of raising the temperature of the extremities." And Dr. CARPENTER considers that, although alcoholic liquors may possibly produce a slight and transitory increase of heat, yet this is but momentary and followed by a depression that more than counterbalances the previous elevation.

Dr. EDWARD SMITH, of London, says:—"In a prolonged inquiry upon myself and another, we took the alcohol in moderate quantity, duly diluted, on an empty stomach, and we noticed most carefully the general effects and the moment of their occurrence. At first, there was a sense of dryness and heat, with fulness or swelling of the exposed parts. After about twenty to forty minutes, this sensation gave place to one of cold, which was first felt on the most sensitive part of the body, with regard to temperature, *viz.*, between the shoulders, and, at length, notwithstanding the existence of a suitable degree of atmospheric temperature, it became distressing, and led even to shivering. This was sometimes so marked, and occurred so suddenly, that it gave rise to a shock."

Dr. SIDNEY RINGER, Professor of Materia Medica, at University College, and Dr. WALTER RICKARDS, in an article in the *London Lancet,* give the results of numerous experiments instituted by them, in which they say:—"In their first experiment, they gave alcohol in large doses to three adults. In two, the temperature was greatly depressed—the depression amounting to 3° Fahrenheit. In the third case, the temperature was but little influenced. The subject of this observation was a confirmed drunkard. Alcohol was also, by them, injected into the recti of two rabbits; in both, the temperature was considerably depressed—the depression amounted to 15° Fahrenheit. In a second experiment, they gave alcohol to eleven persons, in ordinary doses (an ounce of brandy). In eight, the temperature was depressed; in three cases, the temperature was unaffected. Two of these were confessed free drinkers. In conducting these observations, the following precautions were taken:—the persons were kept in bed; all the conditions were kept the same; the thermometer was kept the whole time in the axilla, and the temperature noted every few minutes."

In 1848, MM. DUMEREIL and DUMARQUAY, in making numerous experiments on intoxicated dogs, always found their temperature uniformly reduced.

Two years later, 1850, Prof. N. S. DAVIS, of Chicago, instituted a series of experiments, the results of which showed unmistakably that the presence of only a few ounces of either fermented or distilled drinks, in the human system, was sufficient to produce a positive diminution of temperature. To still further verify these facts, Prof. DAVIS again, on the 6th and 11th days of April, 1867, instituted another series of experiments, which also produced the same results. He found that a strong healthy man, who took four ounces of bourbon whiskey diluted with sweetened water, suffered a depression in temperature. The thermometer fell two degrees in an hour. In the second experiment, where he substituted four ounces of sherry wine for the bourbon whiskey, he found the temperature depressed also, but in a less degree. The depression amounted to one-half degree in one hour.

I might continue, and present other authorities, introduce still more proof, but both time and space forbid; and it does seem to me that I have already presented enough to at least cause us to stop and consider the whys and wherefores before we, by example or sanction, fill every human stomach, whose unfortunate possessor is suffering from shock, with alcohol. I simply desire, in conclusion, to add my own views and experience upon this subject. While I have never instituted any scientific investigations, nevertheless, I have had (during a very extensive surgical experience, both in the army and private practice) a pretty good opportunity of witnessing the effect of this agent upon persons laboring under shock. My experience has been, that alcoholic stimulants are very liable to produce extreme nausea, vomiting, and depression of the vital powers. I have thought that they retarded reaction and increased the depression. So marked have been these results, and so distressing their effects, that I have, long since, avoided their administration. I have substituted hot coffee, hot teas, and hot broth, when obtainable, and have been much better pleased with their effects.

Understand me, I do not advocate the extreme touch not, taste not, and handle not principle, I still claim alcohol as a valuable remedial agent; I believe it well worthy of a place in our materia medica—not as a stimulant, not as food, not as a tonic, *but as an antiseptic;* I believe that its great office is to prevent septimia, and, therefore, that it is a valuable remedy in the various zymotic and suppurative diseases. While I would have it retained in the materia medica, I would also have it labelled on every druggist's shelf as an *acrid, irritant poison,* to be used with much care, in extreme cases, *never, under any circumstances, as a beverage,* BY A PRACTITIONER OF MEDICINE, AT LEAST.

